# Identification of status quo and association rules for chronic comorbidity among Chinese middle-aged and older adults rural residents

**DOI:** 10.3389/fpubh.2023.1186248

**Published:** 2023-06-01

**Authors:** Zijing Yu, Yuquan Chen, Qianhang Xia, Qingru Qu, Tao Dai

**Affiliations:** ^1^Institute of Medical Information/Library, Chinese Academy of Medical Sciences, Beijing, China; ^2^Peking Union Medical College, Beijing, China; ^3^PBC School of Finance, Tsinghua University, Beijing, China

**Keywords:** rural areas, middle-aged and older adults, chronic comorbidity, association rules, chronic disease

## Abstract

**Background:**

Chronic comorbidity has become a major challenge in chronic disease prevention and control. This issue is particularly pronounced in rural areas of developing countries, where the prevalence of chronic disease comorbidity is high, especially among middle-aged and older adults populations. However, the health status of middle-aged and older adults individuals in rural areas of China has received inadequate attention. Therefore, it is crucial to investigate the correlation among chronic diseases to establish a reference basis for adjusting health policies aimed at promoting the prevention and management of chronic diseases among middle-aged and older adults individuals.

**Methods:**

This study selected 2,262 middle-aged and older adults residents aged 50 years or older in Shangang Village, Jiangsu Province, China, as the study population. To analyze the chronic comorbidity of middle-aged and older adults residents with different characteristics, we used the *χ*^2^ test with SPSS statistical software. Data analysis was conducted using the Apriori algorithm of Python software, set to mine the strong association rules of positive correlation between chronic disease comorbidities of middle-aged and older adults residents.

**Results:**

The prevalence of chronic comorbidity was 56.6%. The chronic disease comorbidity group with the highest prevalence rate was the lumbar osteopenia + hypertension group. There were significant differences in the prevalence of chronic disease comorbidity among middle-aged and older adults residents in terms of gender, BMI, and chronic disease management. The Apriori algorithm was used to screen 15 association rules for the whole population, 11 for genders, and 15 for age groups. According to the order of support, the most common association rules of comorbidity of three chronic diseases were: {lumbar osteopenia} → {hypertension} (support: 29.22%, confidence: 58.44%), {dyslipidemia} → {hypertension} (support: 19.14%, confidence: 65.91%) and {fatty liver} → {hypertension} (support: 17.82%, confidence: 64.17%).

**Conclusion:**

The prevalence of chronic comorbidity among middle-aged and older adults rural residents in China is relatively high. We identified many association rules among chronic diseases, dyslipidemia is mostly the antecedent, and hypertension is primarily the result. In particular, the majority of comorbidity aggregation patterns consisted of hypertension and dyslipidemia. By implementing scientifically-proven prevention and control strategies, the development of healthy aging can be promoted.

## 1. Introduction

In recent years, chronic non-communicable diseases (hereinafter referred to as chronic diseases), mainly including cardiovascular and cerebrovascular diseases, cancer, chronic respiratory diseases, and diabetes, have become a significant public health problem worldwide. The causes of chronic diseases are complex, and the course of the disease is prolonged. The negative impact on society, families, and individuals cannot be underestimated (1). WHO has predicted that by 2030, the number of deaths caused by chronic diseases in the world will account for 75% of the total deaths (2). In 2019, the number of deaths caused by chronic diseases in China accounted for 88.5% of the total number of deaths (3). Its incidence and prevalence rates showed an upward trend, and the disease burden caused by chronic diseases accounted for about 70% of the total burden (4). Chronic diseases pose severe challenges to people’s quality of life (QoL), and it is particularly important to carry out the prevention and treatment of chronic diseases.

Ensuring the physical and mental health of the population hinges on effective prevention and control of chronic diseases. However, addressing chronic comorbidity poses a significant challenge in this regard. Chronic comorbidity refers to an individual’s long-term co-occurrence of two or more chronic diseases (5–7). Compared with a single chronic disease, chronic disease comorbidity will worsen and reduce the QoL of the older adults, leading to the decline of patients’ physical function, QoL, disease burden, etc. (8), increasing the difficulty of medical workers’ diagnosis and management, and consuming more medical resources (9, 10). Chronic diseases’ high occurrence and prevalence seriously threaten human well-being and quality of daily life.

However, the chronic comorbidity issue was particularly prominent in rural areas of developing countries due to economic backwardness worldwide. Sakib et al. (11) pointed out that the incidence rate of chronic comorbidity among low-income people was high. Zhang X et al. (12) indicated that chronic disease comorbidity was prevalent in China and India, with median prevalence being 36.1% (IQR 19.6, 48.8) and 28.3% (IQR 8.9, 56.8), and the burden of disease was increasing. Compared with cities, China’s rural health service system is not sound enough, medical technology is relatively backward, and the health level of the population is relatively low. Influenced by behavioral risk factors, residents’ unhealthy diets, and habits of work and resting with high salt, high fat, and polysaccharide are widespread (13). The number of chronic disease patients in China is still expanding, and the prevention and treatment of chronic diseases are imminent. In addition, with the deepening of global aging, the risk of chronic diseases for middle-aged and older adults people is gradually increasing. This specific population is particularly susceptible to chronic diseases, and as a result, chronic disease comorbidity is more prevalent among them (14–18). To sum up, it is significant to study the status quo of chronic disease comorbidity among middle-aged and older adults people in rural areas of China (19, 20).

Nowadays, more countries pay attention to chronic comorbidity, and scholars from many countries have begun to study the association model and population characteristics of chronic comorbidity relying on diverse methodologies (21, 22). Im et al. (23) used a multivariable regression model to determine the correlation of chronic comorbidity. Shi et al. (24) used the Markov chain to estimate the probability of another situation in the next state after diagnosis and then conducted weighted association rule mining. Held et al. (25) used network analysis, cluster analysis, and association rules to analyze chronic diseases. Association rules have become an increasingly popular approach for analyzing medical data in recent years. By applying association rules to chronic disease comorbidities, it is possible to identify patterns of two or more chronic diseases, thereby shedding light on the relationships and associations between multiple chronic diseases. Few studies have clarified the pattern of chronic comorbidity in China, and there are relatively few relevant studies. Consequently, our team has undertaken this mission and attempted to bridge this gap through a representative area of the status quo of chronic comorbidities and potential comorbidities among middle-aged and older adults residents in rural China.

Collectively, this paper aimed to answer the following two key questions:

- What is the status quo of chronic comorbidity among middle-aged and older adults people in rural China?
- What are the causal association rules between different chronic diseases?

## 2. Methods

### 2.1. Data source and indicator determination

This study selected Shangang Village in Jiangsu Province, a well-developed eastern rural area in China, as an example. The data for the study participants were collected from the grassroots medical and health information system of the sample population. The system included the health examination data (name, gender, age, health evaluation, health guidance, physical examination impression, etc.) of all middle-aged and older adults people participating in the basic medical insurance for urban and rural residents in 2020. Select the top five chronic diseases (lumbar osteopenia, hypertension, dyslipidemia, fatty liver, and cholelithiasis) with the highest frequency in the data set, and make statistics and analysis on the prevalence of chronic diseases among the middle-aged and older adults residents and their association rules.

### 2.2. Judgment criteria

According to the diagnostic criteria for primary osteoporosis recommended by WHO, lumbar osteopenia is defined: *T*-value≥ −1.0 SD is diagnosed as normal bone mass, −2.5 SD <*T*-value <−1.0 SD is the low bone density or bone mass reduction, and *T*-value ≤−2.5 SD is osteoporosis (26, 27). The criteria for hypertension are: mean systolic blood pressure ≥140 mmHg and/or diastolic blood pressure ≥ 90 mmHg or antihypertensive drugs were taken in recent 2 weeks. Dyslipidemia: including any one of triglyceride, hypercholesterolemia, high-density lipoprotein, low-high-density lipoprotein, and mixed hyperlipidemia. Fatty liver: confirmed as fatty liver by pathological examination of liver tissue and B ultrasonic examination. Cholelithiasis: There is a stable strong echo light mass in the gallbladder, or it moves with the change of body position, or there is a sound shadow behind it (28). Body mass index (BMI): between 18.5 and 24 is normal, below 18.5 is lean, between 24 and 28 is overweight, and above 28 is obese.

### 2.3. Principle of association rules

The Apriori algorithm was used to mine association rules between chronic diseases in this study. The so-called association rule is an essential data mining technique used to discover the relevant rules between things and reflect the correlation and dependency between things. If there is a certain rule between the values of two or more variables, one of them can be used to predict other variables (29). Among the many effective algorithms in association rule analysis, the Apriori algorithm has the best performance and the widest application. The Apriori algorithm is a classical algorithm of prior probability. It uses the prior knowledge of the nature of frequent itemsets, and exhausts all frequent itemsets in the data set through the iterative method of layer-by-layer search, thus mining the potential links of the data. Applying association rules to chronic comorbidity can explore the pattern of chronic comorbidity and obtain the causal relationship among chronic diseases (30, 31).

Support, confidence, and lift are commonly used metrics for association rules. The specific concepts are as follows: Set D as the sample database and set A and B as the classification variables in the database.


(1) Support(A→B)=P(A∩B)/P(D)

Support indicates the probability of A and B co-occurring in database D. The higher the support, the higher the possibility of A and B co-occurring, and the more critical the association rule is.


(2) Confidence(A→B)=P(B|A)=P(A,B)/P(A)

Confidence refers to the conditional probability that B occurs when A occurs in database D. The higher the confidence level, the higher the confidence level of the association rule.


(3) Lift(A→B)=P(B|A)/P(B)

Lift indicates that in database D when A occurs, the conditional probability of B occurrence is a multiple of the unconditional probability of B occurrence. A high lifting degree stands for the strong impact of A on B and vice versa, which can further reflect the correlation between these two variables in association rules. When the lifting degree is greater than 1 and higher, the higher the positive correlation between A and B is.

These three standards are indispensable, and the absence of any one indicator may lead to incorrect conclusions. The support, confidence, and lift obtained are, respectively, greater than the association rules with the minimum support, minimum confidence, and minimum lift set by the user, called strong association rules.

### 2.4. Statistical analysis

In this study, the statistical software SPSS 25.0 was used to conduct statistical analysis on five selected chronic diseases. Gender, age, body mass index (BMI), smoking, drinking, chronic disease management, and other factors were included, and the *χ*^2^ test was used to analyze the difference in chronic disease comorbidity among middle-aged and older adults residents. The test level was *α* = 0.05. In order to analyze the correlation and correlation strength between chronic diseases, the mlxtend package in Python 3.8 software was used for data mining of the Apriori algorithm of association rules. Set the minimum support to 1.00%, the minimum confidence to 50.00%, and the lift to be greater than 1.00.

## 3. Results

### 3.1. Basic information of respondents

**Figure 1:**
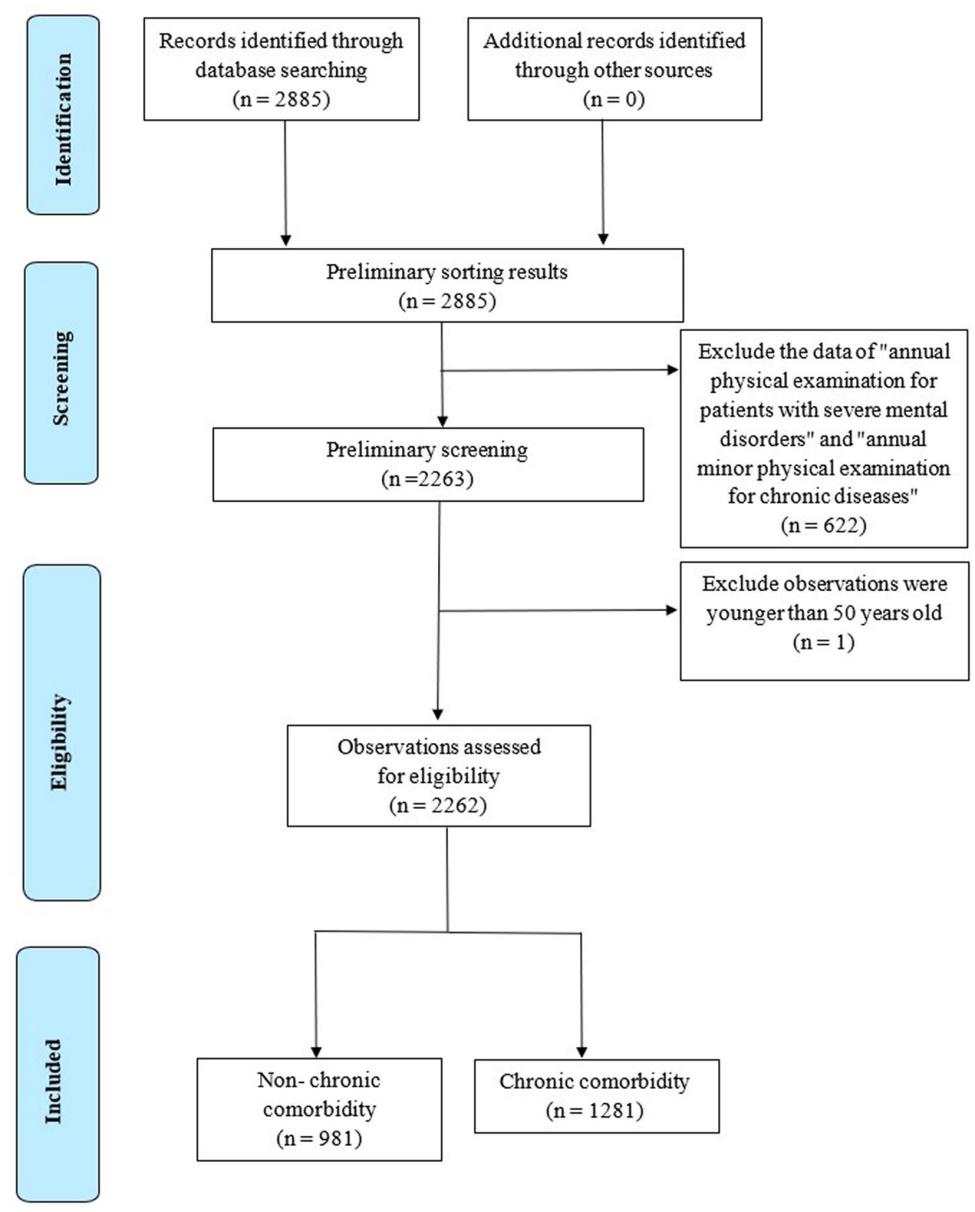
PRISMA flow chart of included studies.

The middle-aged and older adults residents ≥50 years old in the system were selected as the survey objects, and 2,262 pieces of valid data were collected, excluding incomplete information (as shown in Figure 1). The respondents were 35.5% male and 64.5% female, with an average age of 69 years. According to age, 50~59 years old accounted for 18.2%, 60~69 years old for 32.6%, 70~79 years old for 37.2%, and 80 years old and above accounted for 12.0%. The normal rate of BMI was 49.6%, while the rate of smoking, drinking, and chronic disease management was 14.1%, 14.7%, and 48.1%, respectively. The prevalence rate of chronic diseases among middle-aged and older adults residents was 86.7%, including 56.9% of hypertension, 50.0% of lumbar osteopenia, 29.1% of dyslipidemia, 27.8% of fatty liver, and 9.2% of cholelithiasis.

### 3.2. Current situation of chronic comorbidity

The survey results showed that there were 1,281 middle-aged and older adults residents with chronic comorbidity diseases, with a prevalence rate of 56.6%. Among them, the comorbidity rate of two chronic diseases was 33.5%, the comorbidity rate of three chronic diseases was 17.2%, the comorbidity rate of four chronic diseases was 5.7%, and the comorbidity rate of five chronic diseases was 0.3%. The top three disease combinations were:

1. Lumbar osteopenia + hypertension group (310 persons, 13.7%);
2. Lumbar osteopenia + hypertension + dyslipidemia group (117, 5.2%);
3. Hypertension + dyslipidemia group (106, 4.7%).

The lowest prevalence rate was dyslipidemia + cholelithiasis group (2 persons, 0.1%).

**Figure 2:**
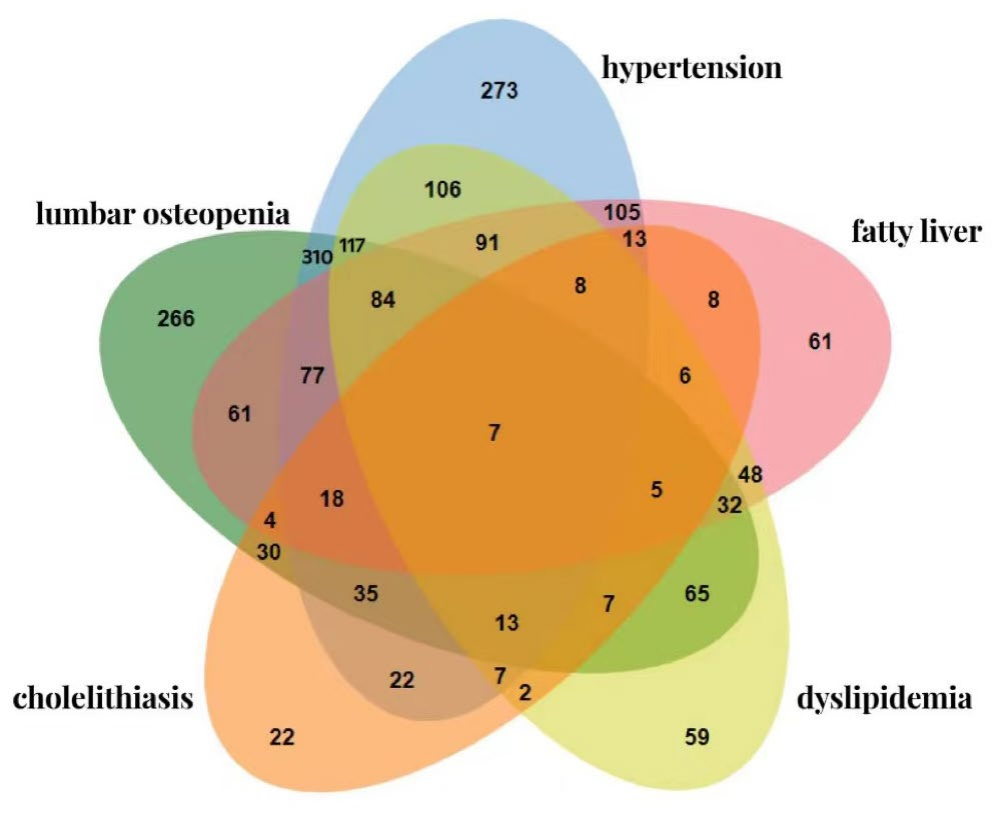
Venn diagram of the prevalence of chronic comorbidity in middle-aged and older adults residents.

The highest prevalence rate of the dual mode was the lumbar osteopenia reduction + hypertension group, the highest prevalence rate of the ternary mode was the lumbar osteopenia + hypertension + dyslipidemia group, and the highest prevalence rate of the four modes was the lumbar osteopenia + hypertension + fatty liver + dyslipidemia group, as shown in Figure 2.

The distribution of chronic comorbidity among middle-aged and older adults residents in the sample area showed that the comorbidity rate of chronic diseases in women (58.6%) was higher than that in men (53.0%), with a statistically significant difference (*χ*^2^ = 6.50, *p* < 0.05). With the increase in BMI, the overall trend of chronic comorbidity was increasing (the trend *χ*^2^ value is 148.76, *p* < 0.05). The middle-aged and older adults residents with BMI in the obesity range had the highest comorbidity of 2, 3, and 4 chronic diseases. The prevalence of chronic comorbidity of obese residents was about 1.8 times that of normal-weight residents. The comorbidity rate of residents included in chronic disease management (46.4%) was lower than that of residents not included in chronic disease management (66.1%), with a statistically significant difference (*χ*^2^ = 88.08, *p* < 0.05). There was no significant difference in the distribution of chronic comorbidity among age, smoking, and drinking (*p* > 0.05) (as shown in Table 1).

**Table 1:** Prevalence of chronic comorbidity among middle-aged and older adults residents.

Features	Number of people	Prevalence^a^						Comorbidity^b^	*χ*^2^ value	*p*-value
		0 kind	1 kind	2 kinds	3 kinds	4 kinds	5 kinds			
Gender									6.50	<0.05
Male	803	122 (15.2)	255 (31.8)	250 (31.1)	131 (16.3)	45 (5.6)	0 (0.0)	426 (53.0)		
Female	1,459	178 (12.2)	426 (29.2)	507 (34.7)	258 (17.7)	83 (5.7)	7 (0.5)	855 (58.6)		
Age									3.19	>0.05
50~59	413	51 (12.3)	102 (24.7)	154 (37.3)	78 (18.9)	27 (6.5)	1 (0.3)	260 (63.0)		
60~69	737	97 (13.2)	223 (30.3)	250 (33.9)	127 (17.2)	39 (5.3)	1 (0.1)	417 (56.5)		
70~79	841	121 (14.4)	260 (30.9)	267 (31.7)	144 (17.1)	45 (5.4)	4 (0.5)	460 (54.7)		
80~	271	31 (11.4)	96 (35.4)	86 (31.7)	40 (14.8)	17(6.3)	1(0.4)	144 (53.2)		
BMI									148.76	<0.05
Lean	72	13 (18.1)	28 (38.9)	23 (31.9)	7 (9.7)	0 (0.0)	1 (1.4)	31 (43.0)		
Normal	1,122	208 (18.5)	401 (35.7)	345 (30.8)	144 (12.8)	22 (2.0)	2 (0.2)	513 (45.8)		
Overweight	792	63 (8.0)	216 (27.3)	270 (34.0)	165 (20.8)	75 (9.5)	3 (0.4)	513 (64.7)		
Obese	276	16 (5.8)	36 (13.0)	119 (43.1)	73 (26.5)	31 (11.2)	1 (0.4)	224 (81.2)		
Smoking status									3.39	>0.05
Smoke	318	48 (15.1)	105 (33.0)	98 (30.8)	52 (16.4)	14 (4.4)	1 (0.3)	165 (51.9)		
No smoking	1944	252 (13.0)	576 (29.6)	659 (33.9)	337 (17.3)	114 (5.9)	6 (0.3)	1,116 (57.4)		
Drinking status									0.36	>0.05
Drink	332	40 (12.0)	99 (29.9)	113 (34.1)	61 (18.4)	17 (5.1)	2 (0.6)	193 (58.2)		
No drinking	1930	260 (13.5)	582 (30.2)	644 (33.4)	328 (17.0)	111 (5.6)	5 (0.3)	1,088 (56.3)		
Chronic disease management									88.08	<0.05
Included	1,088	206 (18.9)	377 (34.7)	314 (28.9)	146 (13.4)	44 (4.0)	1 (0.1)	505 (46.4)		
Excluded	1,174	94 (8.0)	304 (25.9)	443 (37.7)	243 (20.7)	84 (7.2)	6 (0.5)	776 (66.1)		

The data outside the a brackets are examples, and the data inside the brackets are composition ratio (%); the data outside the b brackets are the number of people, and the data inside the brackets are the prevalence rate of comorbidity (%).

**Figure 3:**
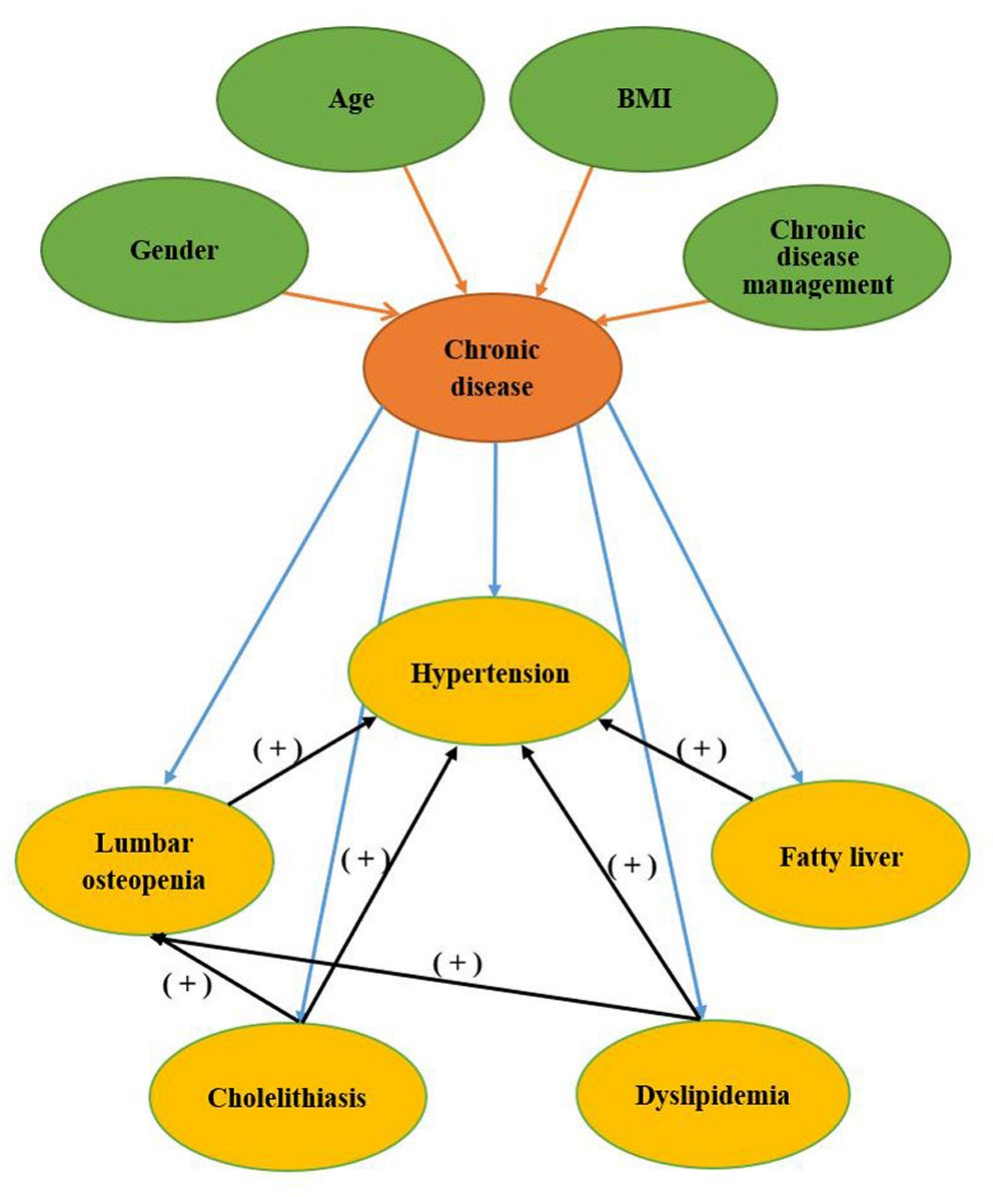
The relationship of high-frequency keywords.

To supplement the quantitative data and further explore the relationship between demographic and sociological characteristics and the prevalence of chronic disease comorbidities among middle-aged and older adults individuals, we randomly selected 40 survey subjects from the database and conducted follow-up interviews. Our interviews revealed the following: (1) Education status—75% of the respondents had a primary school education or below, and we found no significant relationship between education status and chronic disease comorbidities. (2) Monthly net income situation—45% and 50% of the respondents had a monthly net income of less than 1,000 yuan and between 1,000 yuan and 5,000 yuan, respectively, with very few individuals having a net income between 5,000 yuan and 10,000 yuan. Interestingly, as monthly net income increased, the incidence of chronic comorbidities among respondents decreased. During the interviews, several participants mentioned that they did not seek medical attention for minor illnesses due to financial constraints, resulting in their condition worsening over time. (3) Previous medical history—the interviewees had almost no history of genetic diseases or allergies, and about 25% of them had a history of surgery. However, we found no significant relationship between past medical history and chronic comorbidities (see Figure 3).

### 3.3. Analysis of association rules of chronic comorbidity

The results showed that 15 association rules conform to the above settings, which was the comorbidity mode with strong correlation strength. Among them, there were 6 association rules for binary patterns, 7 association rules for ternary patterns, and 2 association rules for quad patterns. Among the 15 association rules, dyslipidemia was the antecedent chronic disease with the highest frequency, and hypertension was the consequent chronic disease with the highest frequency. The latter item only included 2 diseases, including 12 hypertension and 3 lumbar osteopenia (see Figure 3).

The top 3 association rules with the highest support were (as shown in Table 2):

1. {Lumbar osteopenia} → {Hypertension};
2. {Dyslipidemia} → {Hypertension};
3. {Fatty liver} → {Hypertension}.

**Table 2:** Analysis of the association of chronic diseases in middle-aged and older adults residents.

Antecedent	Consequent	Support/%	Confidence/%	Lift
Cholelithiasis	Hypertension	5.44	59.42	1.05
Cholelithiasis	Lumbar osteopenia	5.26	57.49	1.15
Dyslipidemia	Lumbar osteopenia	14.59	50.23	1.01
Dyslipidemia	Hypertension	19.14	65.91	1.16
Fatty liver	Hypertension	17.82	64.17	1.13
Lumbar osteopenia	Hypertension	29.22	58.44	1.03
Cholelithiasis, dyslipidemia	Hypertension	1.55	63.64	1.12
Cholelithiasis, dyslipidemia	Lumbar osteopenia	1.41	58.18	1.16
Cholelithiasis, fatty liver	Hypertension	2.03	66.67	1.17
Cholelithiasis, lumbar osteopenia	Hypertension	3.22	61.34	1.08
Dyslipidemia, lumbar osteopenia	Hypertension	9.77	66.97	1.18
Fatty liver, dyslipidemia	Hypertension	8.40	67.62	1.19
Fatty liver, lumbar osteopenia	Hypertension	8.22	64.58	1.14
Cholelithiasis, fatty liver, lumbar osteopenia	Hypertension	1.11	73.53	1.29
Dyslipidemia, fatty liver, lumbar osteopenia	Hypertension	4.02	71.09	1.25

The top 3 association rules with the highest confidence and lift were the same, which were:

1. {Cholelithiasis, fatty liver, lumbar osteopenia} → {Hypertension};
2. {Fatty liver, dyslipidemia, lumbar osteopenia} → {Hypertension};
3. {Fatty liver, dyslipidemia} → {hypertension}.

The data were analyzed separately according to gender and age group, and 28 and 52 association rules of comorbidity were obtained, respectively. Due to too many association combinations, we increased the promotion degree to more than 1.19 and obtained 11 gender-related and 15 age-group-related association rules. Among the 11 association rules related to gender, the highest support was {dyslipidemia, lumbar osteopenia} → {hypertension} in men. The highest confidence level and lift were {cholelithiasis, fatty liver, lumbar osteopenia} → {hypertension} of women. The number of association rules for chronic diseases in men was less than in women. In the association rules obtained, the latter term included two chronic diseases, hypertension and lumbar osteopenia, and the incidence of hypertension was high, consistent with the whole population’s results. The association rules found in men only include hypertension, which was a chronic disease, while the latter chronic disease in women had a certain reduction in lumbar osteopenia (as shown in Table 3).

**Table 3:** Analysis on the association of chronic disease comorbidity in different genders.

Gender	Antecedent	Consequent	Support /%	Confidence/%	Lift
Male	Dyslipidemia, lumbar osteopenia	Hypertension	9.96	67.80	1.21
	Fatty liver, dyslipidemia	Hypertension	7.22	68.24	1.22
	Fatty liver, lumbar osteopenia	Hypertension	7.85	67.00	1.20
	Dyslipidemia, fatty liver, lumbar osteopenia	Hypertension	3.74	75.00	1.34
Female	Cholelithiasis	Lumbar osteopenia	6.17	63.38	1.26
	Cholelithiasis, fatty liver	Hypertension	2.12	70.45	1.23
	Cholelithiasis, dyslipidemia	Lumbar osteopenia	1.58	65.71	1.31
	Cholelithiasis, hypertension	Lumbar osteopenia	3.84	65.88	1.31
	Cholelithiasis, dyslipidemia, lumbar osteopenia	Hypertension	1.10	69.57	1.21
	Cholelithiasis, fatty liver, lumbar osteopenia	Hypertension	1.44	80.77	1.41
	Dyslipidemia, fatty liver, lumbar osteopenia	Hypertension	4.18	69.32	1.21

Among the 15 association rules related to age group, the latter included 4 diseases involving 9 hypertension, 3 lumbar osteopenia, 2 fatty liver, and 1 dyslipidemia. The highest support was {dyslipidemia} → {fatty liver} in the 50~59 age group. The highest confidence level was {hypertension, cholelithiasis} → {lumbar osteopenia} in the 80~age group. The highest degree of improvement was {fatty liver} → {dyslipidemia} in the 80~age group. Among different age groups, dyslipidemia as the first chronic disease had the highest frequency, and hypertension as the second chronic disease had the highest frequency, consistent with the population’s results. The number of association rules showed an overall upward trend with the increase in age. Only two association rules were found in the 50~59 age group, while six association rules were found in the 70~79 age group. And with the increase of age, the results of chronic disease direction began to diversify (as shown in Table 4).

**Table 4:** Analysis of the association of chronic comorbidity in different age groups.

Age	Antecedent	Consequent	Support/%	Confidence/%	Lift
50~59	Dyslipidemia	Fatty liver	15.98	52.80	1.36
	Cholelithiasis, fatty liver	Hypertension	3.15	81.25	1.53
60~69	Dyslipidemia, fatty liver	Hypertension	8.68	69.57	1.20
	Fatty liver, lumbar osteopenia	Hypertension	8.14	69.77	1.20
	Dyslipidemia, fatty liver, lumbar osteopenia	Hypertension	3.80	77.78	1.34
70~79	Cholelithiasis, dyslipidemia	Fatty liver	1.66	53.85	2.20
	Cholelithiasis, dyslipidemia	Hypertension	2.14	69.23	1.20
	Cholelithiasis, dyslipidemia	Lumbar osteopenia	1.90	61.54	1.24
	Dyslipidemia, fatty liver	Hypertension	8.09	70.83	1.23
	Dyslipidemia, lumbar osteopenia	Hypertension	10.58	71.77	1.24
	Dyslipidemia, fatty liver, lumbar osteopenia	Hypertension	3.80	78.05	1.35
80~	Cholelithiasis	Hypertension	5.90	69.57	1.22
	Cholelithiasis	Lumbar osteopenia	7.38	86.96	1.35
	Fatty liver	Dyslipidemia	9.96	51.92	2.47
	Cholelithiasis, hypertension	Lumbar osteopenia	5.17	87.50	1.36

## 4. Discussion

To the best of our knowledge, this study is the first time to clarify the status quo of chronic comorbidities among middle-aged and older adults residents in rural areas of China and analyze the association rules between chronic comorbidities. The study found that the prevalence of chronic comorbidity among middle-aged and older adults residents in the sample area was 56.6%. The prevalence rate of chronic disease comorbidity among the older adults in the China Health and Retirement Longitudinal Study (CHARLS) database in 2018 was 57.3 and 63.8% of the older adults in Jiangsu Province (32, 33), but 51.6% higher than that among the middle-aged and older adults in Shanghai and 43.7% in Guangdong Province (34, 35). It is preliminarily estimated that the prevalence rate of chronic comorbidity in the sample areas is about the average level in China, which is representative. Compared with other countries, the comorbidity rate of chronic diseases in the sample area was 80.0% lower than that of people over 65 years old in Australia (36), 64.1% lower than that of adults in Spain (37), between 37.9% and 64.4% of adult comorbidity in the United States (38), higher than that of adults over 55 years old reported in the Netherlands (39), and 16.3% higher than the comorbidity rate reported in Singapore (40). The difference in results might be related to the age of the respondents and the number of chronic diseases included in the study. The age of the subjects was higher, and more types of chronic diseases were included, which might mean a higher prevalence of comorbidities (38, 41). After a comprehensive analysis, we preliminarily judge that the situation of chronic disease comorbidities in the sample area is relatively serious. According to the field survey, there is a serious shortage of basic-level health human resources in the sample areas. The community health service station has only 3 to 4 medical staff, so the work burden is heavy, and it is difficult to better assume the responsibility of chronic disease management. Simultaneously, the residents lack medical knowledge. There were many unhealthy lifestyles, such as high salt and high oil diet, and poor hygiene habits, which may be the reasons for the high prevalence of chronic diseases in the sample areas. It is suggested that the government should increase the input of medical resources in rural areas, improve the incentive mechanism for medical personnel, and introduce more grassroots talents. We can strengthen the villagers’ awareness of health management and help them improve their unhealthy lifestyles by carrying out health lectures and distributing knowledge manuals. The pilot areas should further make full use of the function of prevention and treatment of chronic diseases among primary health institutions and enhance the surveillance and intervention of those diseases among vulnerable groups.

As the various chronic disease comorbidity combinations formed, hypertension and lumbar osteopenia comorbidity combinations among middle-aged and older adults residents are the most common, which was different from the highest incidence rate of cardiovascular disease comorbidity among medical insurance beneficiaries aged 65 and above studied by Nguyen et al. (42) in the United States. This study selected rural residents, while the comparison population was urban community residents. The results obtained may be related to the region’s characteristics and the survey object population, such as the poor diet habits of rural older adults residents in the sample area with high salt intake, leading to a high prevalence of hypertension. It may be that the long-term physical labor and lack of adequate nutrient supplements led to the decrease of lumbar osteopenia which become a chronic disease with a high prevalence rate. Based on the results of the status quo among sample areas, the prevalence rate of hypertension was the highest, followed by the prevalence rate of lumbar osteopenia, which should be the focus of prevention and treatment of chronic diseases in this area. It is suggested that the pilot areas should further recognize the pathogenesis, treatment methods, daily prevention, and treatment of these two chronic diseases and take comprehensive multidisciplinary care of patients with chronic diseases to improve the health level of middle-aged and older adults residents.

The results of this study showed that there were significant differences in chronic comorbidity among middle-aged and older adults residents by gender, BMI, and chronic disease management. The prevalence rate of chronic disease comorbidity in the sample area was generally higher in women than in men, the higher the BMI, the higher the comorbidity rate, and the higher the rate of chronic disease not included in chronic disease management than in chronic disease management. The prevalence of chronic comorbidity among women was high, which was consistent with the research results of many scholars in South Korea, Indonesia, and other countries (43–47). This study found through interviews that education and past medical history are not closely related to comorbidities, while economic and other factors may be related to residents’ illnesses, which may be influenced by factors such as interview size limitations and sampling bias. The pilot areas should further consider gender differences, especially taking women as a crucial population, combining taking targeted intervention measures to reduce the prevalence of chronic comorbidities. Simultaneously, the pilot areas should pay more attention to the prevalence of chronic comorbidities among overweight or obese middle-aged and older adults residents, strengthen their nutrition, exercise, and other behavioral interventions, and improve the status of overweight and obesity among residents. Furthermore, chronic disease management should include more patients with comorbidities. The governmental department should optimize the health management strategy for chronic diseases and develop corresponding measures for different comorbidity combinations to enhance the health of residents. In order to alleviate the financial strain on patients, it is advisable to enhance the medical insurance system by allocating more resources to the grassroots level through targeted sampling, thereby gradually redirecting the medical insurance fund to where it is needed the most. In future chronic disease prevention and treatment efforts, relevant personnel can categorize the screening population based on gender, BMI, and chronic disease management to improve patient compliance with medical treatment.

The analysis of the Apriori algorithm results of association rules based on the whole population, different genders, and different age groups can draw the following conclusions:

Hypertension might be a multidirectional chronic disease, and dyslipidemia might be associated with hypertension. There may be two reasons for such a result: on the one hand, the association rule algorithm is based on the prevalence rate. The prevalence rate of hypertension among middle-aged and older adults residents in the sample area is higher than that of other chronic diseases, and the results will tend to be a high prevalence of chronic diseases; On the other hand, dyslipidemia and hypertension are cardiovascular diseases with similar risk factors (48). Therefore, if residents have abnormal blood lipids, their blood pressure can be monitored to prevent hypertension.

Compared with men, women were found more association rules and more complex comorbidity. Gender might be one of the risk factors of comorbidity, which was consistent with the results of *χ*^2^ test conducted in this study, as well as with previous studies such as Hernandez et al. (49), Rojas Huerta et al. (50). With the increase of age, women’s ovarian function declined, estrogen secretion decreased, and osteoporosis was more likely to occur, which might be the reason for the chronic disease of lumbar osteopenia in the latter item of the association rules related to women (51, 52). Therefore, this once again reminds us that gender differences should be taken into account in the management of chronic comorbidity.

There were some differences in association rules of chronic comorbidity among different age groups. Age is an influencing factor of comorbidity. With the increase of age, the physical function of residents is deteriorating, and the comorbidity of chronic diseases is becoming more complex. It is essential to strengthening the management of comorbidity patterns for the older adults. In addition, the rural economic conditions are poor, the children are mostly employed outside, it is challenging to take care of the older adults at home, and there are many older adults residents left behind. It is suggested that the sample areas should strengthen the health care for the older adults left behind, regularly follow up, distribute basic drugs, etc., to reduce the economic burden of older adults patients with chronic diseases.

In recent years, China has accelerated the implementation of healthy rural construction, implemented the policy of helping the poor and farmers, reformed the rural areas, and greatly improved the economic level. However, there are more middle-aged and older adults people in rural areas. The quality of medical and health services still needs to be improved, and the health status of the population is relatively poor. There is still a long way to go to improve residents’ health. It is suggested that the government should increase the input of medical resources, establish a health service mechanism corresponding to the health of the older adults, improve the prevention and health service system, promote the integration of health and physical education, and improve the health level of the middle-aged and older adults by improving public health services (53).

## 5. Limitations

This article analyzed the current situation and association rules of chronic comorbidities among middle-aged and older adults people in Jiangsu Province, a well-developed rural area in China. However, the study also had some limitations, as the respondents may not have covered the entire population over 50 years old. Additionally, the information collected on chronic diseases was not very comprehensive, and some data were obtained through diagnostic conclusions. Furthermore, the categories of chronic diseases included were not complete, which could have affected the results. In future studies, more accurate and comprehensive data should be collected to explore the correlation between chronic comorbidities and provide a scientific basis for preventing and treating chronic comorbidities. Scientific prevention and control measures would undoubtedly reduce the harm caused by chronic comorbidity and help promote the development of healthy aging.

## 6. Conclusion

To sum up, this study found that the overall prevalence of chronic comorbidities among middle-aged and older adults residents in rural China is relatively high, and the situation is relatively tricky. By analyzing the current status of chronic comorbidities, it was found that the prevalence of hypertension and lumbar osteopenia is higher in the sample area. There is a statistically significant difference in the prevalence of chronic comorbidities among middle-aged and older adults residents among different genders, BMI, and chronic disease management methods. Among the association rules formed for chronic diseases, the results of the association rules often point to hypertension. Hypertension and dyslipidemia constitute a majority of comorbidity aggregation patterns, and monitoring the blood pressure status of patients with dyslipidemia has a positive effect on preventing hypertension. In addition, gender and age differences should be given attention in managing chronic disease comorbidities. Improve the health level of middle-aged and older adults people by improving public health services and strengthening the management of chronic disease comorbidities.

## Data availability statement

The raw data supporting the conclusions of this article will be made available by the authors, without undue reservation.

## Ethics statement

Ethical review and approval was not required for the study on human participants in accordance with the local legislation and institutional requirements. The patients/participants provided their written informed consent to participate in this study.

## Author contributions

ZY: conceptualization, data collection, and writing—original draft preparation. ZY and YC: methodology. ZY and QX: data extraction. ZY and QQ: data analysis. ZY, YC, QX, QQ, and TD: writing—review and editing. TD: supervision, project administration, and funding acquisition. All authors contributed to the article and approved the submitted version.

## Conflict of interest

The authors declare that the research was conducted in the absence of any commercial or financial relationships that could be construed as a potential conflict of interest.

## Publisher’s note

All claims expressed in this article are solely those of the authors and do not necessarily represent those of their affiliated organizations, or those of the publisher, the editors and the reviewers. Any product that may be evaluated in this article, or claim that may be made by its manufacturer, is not guaranteed or endorsed by the publisher.
